# Towards a More Realistic In Vitro Meat: The Cross Talk between Adipose and Muscle Cells

**DOI:** 10.3390/ijms24076630

**Published:** 2023-04-01

**Authors:** Margherita Pallaoro, Silvia Clotilde Modina, Andrea Fiorati, Lina Altomare, Giorgio Mirra, Paola Scocco, Alessia Di Giancamillo

**Affiliations:** 1 Department of Veterinary Medicine and Animal Sciences (DIVAS), University of Milan, Via dell’Università 6, 26900 Lodi, Italy; margherita.pallaoro@unimi.it (M.P.); silvia.modina@unimi.it (S.C.M.); 2 Department of Chemistry, Materials and Chemical Engineering “G. Natta”, Polytechnic University of Milan, Via Luigi Mancinelli, 7, 20131 Milan, Italy; andrea.fiorati@polimi.it (A.F.); lina.altomare@polimi.it (L.A.); 3 National Interuniversity Consortium of Materials Science and Technology (INSTM), 50121 Florence, Italy; 4 Department of Comparative Biomedicine and Food Science, University of Padua, Viale dell’Università 16, 35020 Legnaro, Italy; giorgiomirra5@gmail.com; 5 School of Biosciences and Veterinary Medicine, University of Camerino, Via Gentile III da Varano, 62032 Camerino, Italy; paola.scocco@unicam.it; 6 Department of Biomedical Sciences for Health, University of Milan, Via Mangiagalli 31, 20133 Milan, Italy

**Keywords:** in vitro meat, cells co-culture, muscle and adipose cell cross talk, scaffold, hydrogel, farm-animal-derived cells

## Abstract

According to statistics and future predictions, meat consumption will increase in the coming years. Considering both the environmental impact of intensive livestock farming and the importance of protecting animal welfare, the necessity of finding alternative strategies to satisfy the growing meat demand is compelling. Biotechnologies are responding to this demand by developing new strategies for producing meat in vitro. The manufacturing of cultured meat has faced criticism concerning, above all, the practical issues of culturing together different cell types typical of meat that are partly responsible for meat’s organoleptic characteristics. Indeed, the existence of a cross talk between adipose and muscle cells has critical effects on the outcome of the co-culture, leading to a general inhibition of myogenesis in favor of adipogenic differentiation. This review aims to clarify the main mechanisms and the key molecules involved in this cross talk and provide an overview of the most recent and successful meat culture 3D strategies for overcoming this challenge, focusing on the approaches based on farm-animal-derived cells.

## 1. Introduction

Meat and meat-derived products are the most consumed food and they are essential in a balanced diet to provide proteins, essential amino acids, vitamins, iron, zinc and fatty acids [1].

According to the Chatham House Report, today, 50% of the world’s habitable land is occupied by cropping and animal farming [2], while the livestock amounts to approximately 65% of all mammals on Earth [3].

This represents a serious problem since livestock and the food industry are involved in critical environmental issues; according to FAO, livestock is responsible for producing 7.1 gigatonnes of CO2-equivalent per year, which is 14.5% of the total emission of anthropogenic greenhouse gases [4].

As reported by the Global Energy Review–2022, the production of CO2 from the most impacting activities, that are combustion and industrial processes, is about 36.3 gigatonnes [5].

Another critical point is the use of water in the animal-derived food industry, which is the primary agricultural cause of water wastage and pollution, significantly contributing to world water withdrawals and the discharge of toxic compounds such as drug residues and agrochemicals into the groundwater [6].

Additionally, as agriculture and livestock are steadily growing, an increase in both agricultural land use and the related levels of greenhouse gases seems inevitable. This would make farming indirectly responsible for the loss of biodiversity since greenhouse gases play a role in climate change and rising temperatures, the leading causes of wild animals either abandoning their natural habitats or dying [2].

Finally, finding an alternative to animal-derived food consumption could solve several ethical issues related to animal welfare [7].

Considering all the drawbacks linked to livestock and farming activities, different strategies to answer the growing demand for meat have been proposed, from plant-based alternatives to insect flour, low-impact farming, and cultured meat [8].

In vitro meat represents one of the most promising, but at the same time challenging, alternatives to meat. This technique is based on growing cells in specific conditions to obtain a meat-like structure, endowed with meat sensory characteristics. The first attempt at culturing meat was performed in 1971 by Russel Ross, who used pig smooth muscle-derived cells to obtain fibers [9]. However, it was only in 2013 that the team of Professor Mark Post from the University of Maastricht developed the first cultured hamburger [10], opening new perspectives and challenges and paving the way toward modern approaches to in vitro meat culturing. Innovations in cell culturing methods have been applied to this field with good results, especially with the introduction of 3D supports that made obtaining a meat-looking product even more realistic [10,11,12,13]. Despite some of the difficulties involved in co-culturing those cell types that are naturally typical of meat, recent strides have led to promising results, propelling in vitro meat culturing towards new perspectives and development possibilities [1,13,14,15,16].

This review aims to provide an up-to-date overview of novel approaches in this field, examining the critical, technical aspects related to the necessity of obtaining a more realistic in vitro product. This study seeks to examine (i) the cross talk between adipose and muscle cells and (ii) the novel biotechnological approaches to meat culturing, with a specific emphasis on achievements in using 3D supports and large farm-animal-derived cells and their potential in the pursuit of a realistic in vitro meat.

## 2. The Cross Talk between Adipose and Muscle Cells

### 2.1. In Vitro Meat—The Challenge

Muscles are constituted by different tissues identified in meat, including contractile muscle fibers, connective tissue, blood vessels, lymph capillaries, motor endplate, and adipose tissue [17,18,19,20,21,22]. Among these, muscle fiber and connective tissue, including intramuscular fat, are the main ones responsible for meat’s organoleptic characteristics of tenderness and juiciness, together with water [23,24]. These characteristics are evaluated by consumers and determine the quality of meat together with the meat color [18,20,25]. Hence, it has been and still is difficult to recreate those characteristics in an in vitro condition of meat production [1]. One of the main challenges in this process is the co-culturing of different cell types.

Co-culturing is a technique that allows cultivating together different cell types, either directly or indirectly [26]. Briefly, in the direct system, different cell types are cultured spatially together in the same dish, while in the indirect system, different cell populations are cultured separately, for example, using Transwell inserts or specific chambers, and they only communicate via secretory factors [27]. These systems are already widely used for different purposes, such as in studies on the cross talk between different tissues, for example, between endothelial and smooth muscle cells or between microglia and neuronal cells [28,29], and in research on drug delivery [30,31]. However, their application in in vitro meat reproduction is only beginning to find its way.

Ideally, to properly reproduce meat in an in vitro condition, it would be necessary to co-culture muscle cells, adipocytes, nerve, and blood cells [1]. However, current in vitro meat production relies mainly on the co-culture of muscle and fat cells to obtain a good texture and juiciness of the final product. Marbling, which refers to the intramuscular fat between muscle fibers, is the main characteristic responsible for meat juiciness and is an important parameter used to grade meat products [32]. Thus to obtain quality in vitro meat products, several studies relied on the co-culturing of adipose and muscle cells [1,6,11]. However, co-culturing these different cell types has faced challenges because of their mutual influence [33]. Similarly to the in vivo conditions where adipose and muscle tissues are in a physical and functional relationship [34,35], in an in vitro co-culture system, such a cross talk can strongly influence the outcome of the culture itself [26]. Thus, several authors have underlined the influence adipocytes growing near muscle fibers can exert in vitro, mainly impairing myogenesis [36,37,38,39,40,41,42].

### 2.2. Adipose and Muscle Cells—The Cross Talk

Research on the molecular mechanisms at the base of this cross talk is mainly performed using rodent or human lineage cells. This step is necessary for obtaining basic information to develop a model for in vitro meat using farm animal cells. Table 1 below schematically summarizes the reported literature.

The existence of such a cross talk could lean on the common embryological mesodermal origin of both skeletal muscles and adipose tissue [42,43]. They both result from mesenchymal precursors, which may explain their strong relationship and level of mutual influence [26], detectable in both in vivo and in vitro conditions.

Seo et al. [36] investigated this cross talk by co-culturing preadipocytes with murine muscle cells. Murine preadipocytes 3T3-L1 were cultured and differentiated on inserts for up to 10 days; then, inserts were placed in 24-well cell culture companion plates where murine myoblasts were seeded the day before starting co-culture. The co-culture was carried out for 5 days prior to analyses. The authors observed a paracrine cross talk between the two cell types, with downregulating effects of adipocytes on the differentiation of myoblasts mainly related to a downregulation in myogenin and upregulation in myostatin, atrophy-related ubiquitin E3 ligases (Atrogin1) and muscle RING-finger protein-1 (MurF-1) in muscle cells. Myogenin is a basic helix–loop–helix transcription factor that belongs to the myogenesis regulatory factors (MRFs) family. It is activated by the myoblast determination protein 1 (MyoD), and it plays a role in myocyte fusion and contractile protein synthesis during myogenesis [44,45,46]. Myostatin, also known as growth differentiation factor 8 (GDF8), is an adipomyokine produced by muscle cells to counteract muscle growth by indirectly activating the protein mothers against decapentaplegic homolog 3 (Smad3), a key element in the transforming growth factor beta pathway which inhibits myogenesis [37,47,48]. Smad3 binds to the basic helix–loop–helix (bHLH) region of MyoD so that MyoD cannot bind the DNA response element enhancer box (E-Box) CAXXTG and consequently cannot activate myogenin expression [49]. The myostatin pathway also leads to an overexpression of the ubiquitin–proteasome system [50], in particular of atrophy-related ubiquitin E3 ligases (Atrogin1) and muscle RING-finger protein-1 (MurF-1) by binding the Activin type II receptor (ActRIIB) [51]. Atrogin1 and MuRF-1 are tissue-specific proteins, typically expressed and upregulated in the proteasome machinery during muscle wasting [51,52,53]. They are responsible for the proteolysis that leads to muscle atrophy [53,54], a condition of skeletal muscle loss to which the main contributor is the ubiquitin–proteasome proteolytic system [55]. Moreover, Seo et al. detected an increased adipomyokine Interleukin-6 (IL-6) level in the murine C2C12 myoblasts. In the study, adipocyte-induced IL-6 played an inhibitory role in muscle cell differentiation [36].

A similar effect of IL-6 on muscle differentiation was also observed by Pelosi et al., who demonstrated that the regulation of muscle differentiation is dependent on the activation of signal transduction cascades with the complex involvement of several kinases [56].

The effects of myostatin on the faith of multipotent cell differentiation were also assessed by Artaza et al. [39], who demonstrated that 10T(1/2) murine mesenchymal multipotent cells stimulated by 5′-azacytidine for myogenic differentiation could undergo such lineage according to the presence or absence of myostatin [39]. They tested two different culture conditions, one characterized by the presence of recombinant myostatin and the other one by the presence of recombinant anti-myostatin. In the first situation, they observed that the levels of MyoD and myogenin were downregulated, with a consequently lower myoblasts fusion index. Interestingly, a higher level of the CCAAT/enhancer-binding proteins alpha (C/EBP alpha) and adiponectin were also recorded, which meant that cells did not undergo the myogenic differentiation pathway in favor of the adipogenic one [39]. C/EBP alpha and the peroxisome proliferator-activated receptor gamma (PPARγ), a key lipid metabolism and energy balance factor, are essential for activating the genes involved in the terminal phase of the adipogenic differentiation [57]. On the other hand, adiponectin is an adipokine that, when upregulated, interacts with PPARγ entering the adipogenesis pathway, as demonstrated on murine preadipocytes by Yang et al. [58]. In the second condition, with the recombinant anti-myostatin supplementation, cells underwent myogenic differentiation, with high levels of MRFs and the absence of adipogenic markers. The study proved that myostatin could interfere with myogenic differentiation, reverting it towards the adipogenic one [39].

Interestingly, Takegahara et al. [40] observed a different influence on myogenesis when using mouse preadipocytes and mature adipocytes. Using an indirect co-culture system based on insert, they cultured together either freshly isolated rat 2G11 mature adipocytes or rat skeletal muscle progenitors. When compared, the two conditions gave different results; using mature adipocytes, a lower fusion index in myoblasts was recorded, with a lower positivity to myosin heavy chains, while using preadipocytes, both the fusion index and the positivity to myosin heavy chains were higher [40]. Moreover, considering the lack of direct contact between the two cell types, the cross talk between them appeared to be mediated by soluble factors. To prove this hypothesis, freshly isolated skeletal muscle progenitors from rat were cultured in two different conditioned media (CMs), one from a 2G11 rat mature adipocytes culture and the other from 2G11 rat preadipocytes [40]. Similar to the co-culture conditions, myogenesis was impaired or slowed when using the mature adipocytes conditioned medium (CM). These results may suggest an effect of myogenesis impairment only when using already differentiated adipose cells.

Accordingly, Choi et al. [41] demonstrated the effects of differentiated bovine adipocytes on bovine muscle cells. Starting from freshly isolated bovine skeletal muscle satellite cells and subcutaneous preadipocytes, cells were separately cultured and differentiated into myoblasts and adipocytes, respectively. Cells were then indirectly co-cultured, seeding adipocytes on an insert and myoblasts on a multi-well plate. Significant differences were found comparing the co-cultured myoblasts with the control monoculture of myoblasts. The co-cultured cells reported a higher expression of both C/EBP beta and PPARγ, both involved in the activation of adipogenesis, as observed by Jin et al. [57]. Choi et al. also observed some differences between adipocytes in the co-culture and the ones used as monoculture controls. Indeed, it showed that the level of G-protein-coupled receptor [48] (GPR43) in the co-cultured group was notably increased [41]. GPR43 is a cell surface receptor widely expressed in adipocytes that regulate metabolic processes and homeostasis [59]. It is responsible for the activation of AMP-activated protein kinase alpha (AMPK alpha), which blocks energy-consuming processes, such as fatty acid biosynthesis, to promote those that produce energy, such as fatty acid oxidation [59,60]. It could be stated that adipocytes co-cultured with myoblasts may lead to ineffective lipid synthesis and increased oxidation [41].

The effects of mature adipocytes co-cultured with satellite cells were also assessed in poultry by Guo et al. [42]. Muscle satellite cells and adipocytes were isolated from chicken *Pectoralis major* muscle. An indirect co-culture using a Transwell insert was set up, with adipocytes seeded on the insert and muscle satellite cells on the lower wells. Analyses revealed that satellite cells in the co-culture showed downregulation in myosin heavy chains (MHC) expression and an accumulation of lipid depots when compared with the control. Moreover, consistent with the reduction in MHC, their expression of myogenin, MyoD, and paired box protein 7 (PAX7), main factors in the myogenic pathway [46,61,62,63], was also reduced [42]. On the other hand, the notable deposition of lipid drops in the co-cultured satellite cells could be explained by the assessed over-expression of genes involved in the PPAR-gamma signaling pathway involved in late adipogenesis [38,56].

**Figure 1:**
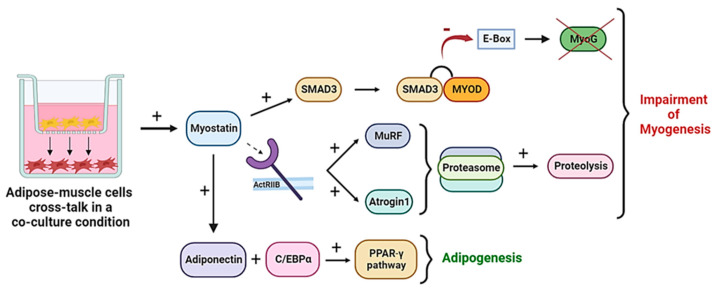
The effects of the cross talk between adipose and muscle cells. In an adipose-muscle cells co-culture, myostatin increases and activates Smad3, which blocks the activity of MyoD and the activation of myogenin (MyoG). Simultaneously, myostatin activates Atrogin1 and MurF-1, triggering proteolysis of muscle proteins. Moreover, adiponectin and C/EBP α, which are important factors of the (PPARγ) pathway, get activated and start adipogenic processes. The final effect of this cross talk is the impairment of myogenesis and a triggered adipogenesis.

In light of these studies, the main effects exerted by adipose cells on muscle cells in a co-culture condition are myogenesis impairment and adipogenesis triggering, mainly due to the overexpression of myostatin, as shown in Figure 1. In summary, myostatin indirectly activates Smad 3 which binds MyoD. This way, MyoD cannot bind E-Box CAXXTG, resulting in a lack of activation of myogenin with consequent impairment of myogenesis. At the same time, the produced myostatin binds the cellular receptor ActRIIB, leading to the production of Atrogin1 and MurF-1, key elements of the proteasome machinery. The consequently triggered proteolysis takes part in myogenesis inhibition by degrading essential muscle proteins. Moreover, the increased level of myostatin is associated with increased adiponectin and C/EBP α, which are factors of the (PPARγ) pathway, which leads to the activation of adipogenic processes. The final effect of this cross talk is an impairment of myogenesis and a triggered adipogenesis.

The downregulation of myogenesis and muscular development was also observed in in vivo conditions, both in the physiological event of aging and under some pathological conditions, such as sarcopenia and obesity [64]. Though such cross talk between adipose and muscular tissue is still to be clarified, it is known that aging and pathologies lead to an inflammatory status for the tissues that, in response, become dysfunctional [65]. This results in altered production of cytokines, adipokines, myokines, and adipomyokines that cross talk, interfering and leading to reciprocal effects [66].

The existence of the cross talk between adipocytes and myocytes may represent a challenge when setting up a co-culture for in vitro meat production. However, thanks to advanced biotechnology and innovative cell culture strategies, research has moved towards effective solutions, such as the application of 3D scaffolds that can improve cultural conditions giving positive outcomes for the pursuit of a more realistic in vitro meat.

## 3. New Approaches and Strategies for In Vitro Meat Culturing

**Figure 2:**
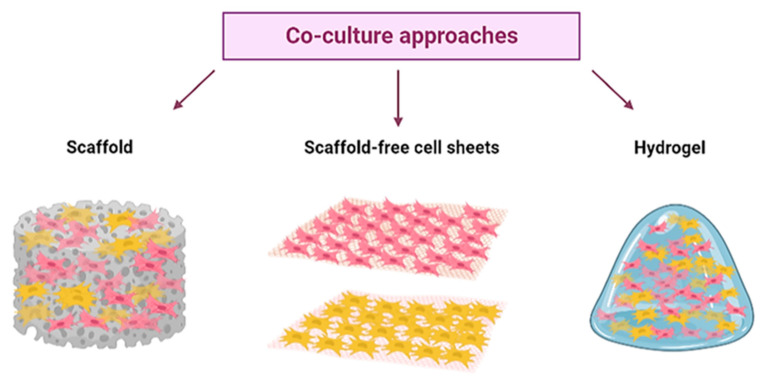
The most recent three-dimensional co-culture approaches to meat agriculture.

The organization of cells for cultured meat production can be achieved by either a scaffolding method or a cell assembly method. As shown in Figure 2, the most recent approaches to 3D meat culture rely on using three different structures: scaffolds, scaffold-free cellular sheets, and hydrogels, as reviewed by Singh et al. [67].

Briefly, a scaffold is a 3D support, generally realized with biomaterials, able to promote cell adhesion, proliferation, and development in a tissue-like structure [68]. To be considered effective, scaffolds should be characterized by properties such as a wide surface and good porosity to promote cell adhesion, proliferation, and differentiation [69] and exhibit a biocompatible and noncytotoxic behavior to guarantee cell survival [70]. Scaffolds are widely used in tissue engineering and regenerative medicine [71], and their application is now finding its way into the field of cultured meat [72,73]. An ideal scaffold for in vitro meat production must be edible and nutritious and show mechanical properties in line with the desired texture for the final products to provide the optimal three-dimensional framework for obtaining reliable and realistic in vitro meat [67,68].

A scaffold-free cell sheet is an approach in which cells are cultured as sheets where the produced extracellular matrix keeps cells aggregated, forming a sort of veil. The cell sheets can be moved and stacked, forming 3D tissue-like structures useful for tissue engineering and meat culturing [74].

Finally, a hydrogel is a three-dimensional support for cell culture, characterized by a high water content, particularly appreciated for its resemblance to the extracellular matrix and for its ability to encourage cell adhesion [75]. A hydrogel shows tissue-like elasticity thanks to its hydration level; moreover, its structure and composition can be modified according to the desired chemical, physical, and biological characteristics [76]. For these reasons, hydrogels have found several practical applications in tissue engineering and are now employed in the meat culturing field. Table 2 schematically summarizes the relevant literature.

### 3.1. Scaffold: Edible Scaffolds

Scaffolds can be realized following several different approaches according to the desired architecture of the final product. Focusing specifically on meat culture, plant-based scaffolds have drawn attention as a possible edible solution for in vitro meat manufacturing [67]. There are several advantages to using vegetable scaffolds; they are biodegradable, cheap, and nutritious and provide a favorable environment for cell adhesion and growth [67]. Their application in cultured meat has already been explored; for example, Thyden et al. produced a scaffold using decellularized broccoli florets on which primary bovine satellite cells were seeded and successfully cultured in a suspension-style bioreactor [77].

Song et al. investigated the possibility of expanding porcine adipose-derived mesenchymal stromal cells (ADSCs) by inoculating them on peanut wire-drawing protein (PWP) scaffolds. They obtained differentiated mature fat, engineered tissue with an improved composition in terms of volatile products compared with the control scaffold-free culture [78].

In addition, wheat gluten was applied in manufacturing 3D scaffolds for cultured meat [79] because it lends itself to producing nontoxic scaffolds with different pore sizes and densities. Xiang et al. investigated the potential applicability of this biomaterial in the production of scaffolds for muscle cell culturing. By adjusting the pore size and the mechanical properties known to affect cell adhesion and proliferation, the authors obtained a wheat gluten scaffold to promote bovine satellite cell proliferation and migration inside the 3D structure [79].

Though the application of scaffolds in cultured meat is promising, to the best of our knowledge, no co-culture of adipose and muscle cells was performed using this kind of support. It is reasonable to state that a cross talk between these two cell populations results in practical issues when realizing a co-culture using scaffolds. Further improvements are required to bypass the inhibition exerted by adipose cells on muscle cell development and make the application of scaffolds, particularly the edible ones, feasible in meat agriculture.

### 3.2. Scaffold-Free Cell Sheets

In 2022, Shahin-Shamsabadi et al. [17] proposed, for the first time, an interesting approach involving the production of layered bio-fabricated cellular sheets. The authors used murine cells as a proof-of-concept model, specifying that this blueprint represents only the preliminary assessment for future farm animal cell development. Shahin-Shamsabadi’s technique was based on producing cellular sheets of muscle and adipose cells starting from partially differentiated murine 3T3-L1 adipocytes and murine C2C12 myoblasts that completed their differentiations co-cultured together in a bi-dimensional condition. The obtained sheets were removed through a delamination process promoted by consequent pH variation and were finally overlapped, forming a meat-like structure [17]. The main advantage of this approach was the lack of a scaffold since the sheets were made stable and robust by the extracellular matrix (ECM) produced by cells. As highlighted by the authors, this approach needs further realization, using, for instance, bioreactors and automated handling to make it suitable for large-scale in vitro meat production [17]. Although some improvements are needed, this sheet model represents a promising opportunity for meat culturing. Indeed, this approach has already been adapted and used for in vitro meat-like structures, using not only murine cells but also bovine ones [79,80]. To our knowledge, the only application of farm animal cells using this strategy was performed by Tanaka et al. [80], who applied the monoculture of freshly isolated bovine myocytes on a scaffold-free cell sheet. The authors used temperature-responsive culture dishes (TRCDs) to obtain cell sheets, and these cultural plates were characterized by a poly(N-isopropylacrylamide) coating, a thermo-responsive polymer, hydrophilic below 32 °C and hydrophobic at 37 °C [82,83,84]. At 37 °C, cells were attached and proliferated during culturing. Then, by lowering the temperature below 32 °C and using ultrasonic washing, the cell sheet detached without degenerating, thanks to the produced ECM. The layers were then layered and cultured to guarantee their attachment and staking. The final result was a meat-like structure composed of piled cellular sheets [80]. Though the authors did not perform a co-culture, their work represents the first employment of large animal cells to the scaffold-free cell sheet approach. Taken together, the works of Tanaka et al. and Shahin-Shamsabadi et al. represent a solid base for applying the cell sheet technique to meat cultured using two different cell types.

### 3.3. Hydrogel

#### 3.3.1. Hydrogel Sheets

Unlike Shahin-Shamsabadi et al., who used no support [17], Li et al. fabricated soy milk gelatin sheets as a culturing matrix. Exploiting the staking-sheets approach, they demonstrated the possibility of generating a solid meat-like structure by consecutively piling three muscle–adipose layers and two adipose layers [81]. The choice of a support made of gelatin and soy milk was made according to the advantages these materials can guarantee. On the one hand, gelatin is rich in integrin-binding sites that promote cell adhesion and migration and favors cell settlement in the support [71,85]. On the other hand, soymilk was chosen for its bioactive isoflavones content that can promote myogenesis by activating myoblast determination protein 1 (MyoD) expression via protein kinase B (Akt or PKB) and p38 pathway [86], and bioactive compounds, such as 6-hydroxydaidzein and protein peptic hydrolysate, that stimulate adipogenesis by increasing the expression of PPAR gamma gene and so promoting lipid accumulation [87,88,89]

#### 3.3.2. Fibrillar Hydrogel

Kang et al. implemented an innovative system based on the assembly of 3D-printed muscle, fat, and endothelial gelatin fibers [16]. Muscle satellite cells and adipose-derived stem cells were isolated from bovine masseter samples. After culturing, their respective differentiation into muscle and adipose and endothelial cells was performed in a 3D bio-printed fibrillar hydrogel of edible gelatin or gellan gum using a supporting bath-assisted 3D printer (SBP) [16]. Moreover, to avoid the collapsing of the fibers into a globular structure, Kang et al. introduced tendon gels to guarantee the linearity of cell fibers during culturing; they named this approach tendon-gel integrated bioprinting (TIP). Once cells were appropriately differentiated, the fibers were physically assembled into a meat-like structure, which was made even more resistant thanks to the addition of transglutaminase, an edible enzyme used as a cross-linker in the food industry [16].

#### 3.3.3. Hydrogel Beads

Zagury et al. [82] also obtained a meat-like structure by engineering bovine adipose tissue; mesenchymal stromal cells were obtained from bovine fat tissue and cultured in alginate hydrogel beads for differentiation. Once the differentiation was reached, integrating adipose cells with muscle cells was performed following two different approaches [82]. On the one hand, differentiated adipose alginate beads were cut and bound through chelation with muscle cells differentiated on freeze-dried alginate scaffolds obtained as previously described by Ben-Arye et al. [73]. On the other hand, adipose cells were extracted from the alginate beads by dissolving the hydrogel and then loading through chelation on alginate-pea protein 3D-printed fibers, on which bovine satellite cells had previously been cultured and differentiated, according to the technique previously developed by Ianovici et al. [90]. Zagury et al. demonstrated the potentiality of chelation as a way to obtain a solid 3D meat-like structure [82]. Moreover, the authors evaluated the dimensions of the final construct and observed better cell maturation when using alginate. This result represents an interesting strategy for extensive cell expansion prior to differentiation to yield a high volume of cellular material starting from limited tissue samples [82].

#### 3.3.4. Gelatin Microcarrier

Finally, among the newest approaches scientists are attempting in order to obtain in vitro meat, the edible 3D porous gelatin microcarrier (PoGelat-MCs) by Liu et al. [15] must be cited. Microcarriers are mini 3D scaffolds endowed with a high surface-to-volume ratio that can be modified for culturing meat purposes to become biodegradable, edible, cell-adhesive, and able to provide a large cultured surface [91], as in the case of the developed PoGelat microcarrier [15]. Freshly isolated pig skeletal muscle progenitor cells and murine 3T3L1 preadipocytes were separately differentiated in flasks; the obtained muscle and adipose cells were seeded and cultured on PoGelat microcarriers into the spinner–flask bioreactor to obtain muscle and adipose microtissues. Once gained, they were assembled in 3D-printed spherical molds where the addition of transglutaminase supported their adhesion into a meatball-like product [15]. Since this only represented a trial, further developments using large animal adipose cells are required to obtain a consumer-acceptable product.

Scaffold-free cell sheets and hydrogels are promising approaches to manufacturing in vitro meat; however, the limitations of such techniques stem from the fact that the co-culture could begin only when the two differentiation processes have already been carried out separately. Indeed, the two cultures are combined and cultured together only at the end of the process to guarantee a successful formation of a 3D meat-like structure thanks to the formation of ECM.

## 4. Conclusions

In vitro meat could represent an ethical and sustainable solution to the increasing demand for meat products. Different attempts to reproduce meat tenderness and juiciness in vitro have been performed by co-culturing those cell types that are typical of muscles. For this purpose, muscle and adipose cell co-culturing strategies have been followed, leading to preliminary studies on the cross talk between cell populations. It has been demonstrated that the main effects of muscle–adipose cell cross talk are the impairment of myogenesis and the triggering of adipogenic processes, which inevitably lead to practical difficulties in performing a successful co-culture. For this reason, it is essential to consider the molecular mechanisms at the base of such cross talk to appoint a co-culture strategy that overcomes myogenesis inhibition. It has been observed that the effects of adipose–muscle cross talk can be successfully overtaken when co-culturing already differentiated cells: it is likely that the strong influence exerted by adipose cells on muscle ones tends to increase or weaken according to the cell differentiation stage. Thus, the most successful approaches in in vitro meat production have involved the use of already differentiated cells and 3D supports in co-cultures, paving the way toward a more realistic product. Edible scaffolds are promising solutions since they are economical, natural, non-impactful, nutritious, and safe. However, to our knowledge, they have been applied only in monoculture, and further developments are likely required to make them functional to host a co-culture. On the other hand, scaffold-free cell sheets and hydrogels were successfully employed in co-culture strategies and could thus represent an optimal solution for meat culture. Moreover, employing large-animal-derived cells for such support makes obtaining reliable in vitro meat even more exciting and successful. Thus far, the results are promising, but further developments and improvements are still required to obtain a well-standardized protocol applicable to large-scale production. Moreover, some challenges still need to be overcome to improve cultured meat manufacturing; above all, there is a necessity to identify the most suitable species and the most appropriate age as optimal sources for cell sorting, since these can be crucial variables that are able to affect the outcome of the process.

## Figures and Tables

**Table 1:** The literature on the in vitro cross talk between adipose and muscle cells.

Authors	Cell Types	Culture System	Outcome	Doi	Year
Seo et al. [36]	3T3-L1	Indirect co-culture	Inhibition of muscle cells differentiation	10.1111/ asj.13145	2019
	C2C12				
Artaza et al. [39]	10T(1/2)	Monoculture:		10.1210/en.2005-0362	2005
		+Recombinant myostatin	Adipogenic differentiation		
		+Recombinant anti-myostatin	Myogenic differentiation		
Takegahara et al. [40]	Rat muscle progenitors	Indirect co-culture:		10.1016/j.yexcr.2014.03.021	2014
	2G11 rat preadipocytes	Preadipocytes + muscle progenitors	No inhibition of muscle differentiation		
	2G11 rat mature adipocytes	Mature adipocytes + muscle progenitors	Inhibition of muscle differentiation		
Choi et al. [41]	Bovine satellite cells	Indirect co-culture	Inhibition of muscle differentiation	10.1016/j.jnutbio.2012.01.015	2013
	Bovine preadipocytes				
Guo et al. [42]	Chicken satellite cells	Indirect co-culture	Inhibition of muscle differentiation	10.1186/s12864-018-5209-5	2018
	Chicken intramuscular adipocytes				

**Table 2:** The most recent and innovative approaches to in vitro meat production.

Support	Author	Species	Cell Types	Approach	Doi	Year
Scaffold - Edible scaffold	Thyden et al. [77]	Bovine	Satellite cells	Decellularized broccoli floret + Rotating bioreactor	10.3390/app12105155	2022
	Song et al. [78]	Pig	Adipose Mesenchymal Stromal Cells	Peanut Wire-drawing Protein scaffold	10.1016/j.foodres.2022.111636	2022
	Xiang et al. [79]	Mouse	C2C12	Wheat gluten scaffold	10.1016/j.biomaterials.2022.121543	2022
		Bovine	Satellite cells			
Scaffold-free cell sheets	Shahin-Shamsabadi et al. [17]	Mouse	3T3-L1	ECM-free bio-fabricated cellular sheets	10.1159/000511764	2022
			C2C12			
	Tanaka et al. [80]	Bovine	Myoblasts	Scaffold-free cell-based sheets	10.1038/s41538-022-00155-1	2022
Hydrogel sheets	Li et al. [81]	Mouse	3T3-L1	Soy milk gelatin sheets	10.3389/fbioe.2022.875069	2022
			C2C12			
Fibrillar Hydrogel	Kang et al. [16]	Bovine	Adipose Mesenchymal Stromal Cells	Bath-assisted 3D printing + tendon gel integrated bioprinting	10.1038/s41467-021-25236-9	2021
			Satellite cells			
Hydrogel beads	Zagury et al. [82]	Bovine	Adipose Mesenchymal Stromal Cells	Alginate spherical Hydrogel	10.1038/s42003-022-03852-5	2022
			Satellite cells	Alginate spherical Hydrogel		
			Adipose Mesenchymal Stromal Cells	Alginate spherical Hydrogel		
			Satellite cells	3D-printed support		
Gelatin microcarrier	Liu et al. [15]	Mouse	3T3-L1	Microcarrier + spinner flasks Bioreactor + Mold	10.1016/j.biomaterials.2022.121615	2022
			C2C12			
		Pig	Satellite cells			

## Data Availability

Not applicable.
